# Dysregulation of Circular RNAs in Myotonic Dystrophy Type 1

**DOI:** 10.3390/ijms20081938

**Published:** 2019-04-19

**Authors:** Christine Voellenkle, Alessandra Perfetti, Matteo Carrara, Paola Fuschi, Laura Valentina Renna, Marialucia Longo, Simona Baghai Sain, Rosanna Cardani, Rea Valaperta, Gabriella Silvestri, Ivano Legnini, Irene Bozzoni, Denis Furling, Carlo Gaetano, Germana Falcone, Giovanni Meola, Fabio Martelli

**Affiliations:** 1 Molecular Cardiology Laboratory, IRCCS Policlinico San Donato, San Donato Milanese, 20097 Milan, Italy; christine.voellenkle@grupposandonato.it (C.V.); alessandra.perfetti@grupposandonato.it (A.P.); carrara.matt@gmail.com (M.C.); paola.fuschi@grupposandonato.it (P.F.); marialucia.longo@grupposandonato.it (M.L.); 2 Laboratory of Muscle Histopathology and Molecular Biology, IRCCS Policlinico San Donato, San Donato Milanese, 20097 Milan, Italy; lauravalentina.renna@grupposandonato.it (L.V.R.); rosanna.cardani@grupposandonato.it (R.C.); giovanni.meola@unimi.it (G.M.); 3 Center for Translational Genomics and Bioinformatics, IRCCS San Raffaele Scientific Institute, 20132 Milan, Italy; baghaisain.simona@hsr.it; 4 Research Laboratories, IRCCS Policlinico San Donato, San Donato Milanese, 20097 Milan, Italy; rea.valaperta@grupposandonato.it; 5 Department of Geriatrics, Orthopaedic and Neuroscience, Institute of Neurology, Catholic University of Sacred Heart, Fondazione Policlinico Gemelli, 00168 Rome, Italy; Gabriella.Silvestri@unicatt.it; 6 Department of Biology and Biotechnology “Charles Darwin”, Sapienza University of Rome, 00185 Rome, Italy; Ivano.Legnini@mdc-berlin.de (I.L.); irene.bozzoni@uniroma1.it (I.B.); 7 Sorbonne Université, INSERM, Association Institut de Myologie, Centre de Recherche en Myologie, F-75013 Paris, France; denis.furling@upmc.fr; 8 Laboratory of Epigenetics, Istituti Clinici Scientifici Maugeri, 27100 Pavia, Italy; carlo.gaetano@icsmaugeri.it; 9 Institute of Cell Biology and Neurobiology, National Research Council, Monterotondo, 00015 Rome, Italy; germana.falcone@cnr.it; 10 Department of Neurology, IRCCS Policlinico San Donato, San Donato Milanese, 20097 Milan, Italy; 11 Department of Biomedical Sciences for Health, University of Milan, 20133 Milan, Italy

**Keywords:** circular RNA, alternative splicing, muscular dystrophies

## Abstract

Circular RNAs (circRNAs) constitute a recently re-discovered class of non-coding RNAs functioning as sponges for miRNAs and proteins, affecting RNA splicing and regulating transcription. CircRNAs are generated by “back-splicing”, which is the linking covalently of 3′- and 5′-ends of exons. Thus, circRNA levels might be deregulated in conditions associated with altered RNA-splicing. Significantly, growing evidence indicates their role in human diseases. Specifically, myotonic dystrophy type 1 (DM1) is a multisystemic disorder caused by expanded CTG repeats in the *DMPK* gene which results in abnormal mRNA-splicing. In this investigation, circRNAs expressed in DM1 skeletal muscles were identified by analyzing RNA-sequencing data-sets followed by qPCR validation. In muscle biopsies, out of nine tested, four transcripts showed an increased circular fraction: CDYL, HIPK3, RTN4_03, and ZNF609. Their circular fraction values correlated with skeletal muscle strength and with splicing biomarkers of disease severity, and displayed higher values in more severely affected patients. Moreover, Receiver-Operating-Characteristics curves of these four circRNAs discriminated DM1 patients from controls. The identified circRNAs were also detectable in peripheral-blood-mononuclear-cells (PBMCs) and the plasma of DM1 patients, but they were not regulated significantly. Finally, increased circular fractions of RTN4_03 and ZNF609 were also observed in differentiated myogenic cell lines derived from DM1 patients. In conclusion, this pilot study identified circRNA dysregulation in DM1 patients.

## 1. Introduction

Myotonic dystrophy type 1 (DM1), also known as Steinert disease (OMIM #160900), is an autosomal dominant multi-systemic disorder with a spectrum of clinical manifestations that includes myotonia, reduced muscle strength, cardiac arrhythmia, insulin resistance, cataracts, hypogonadism, and, in the most severe forms, cognitive defects [1,2,3,4]. The genetic defect in DM1 results from the dynamic expansion of CTG repeats in the 3′ untranslated region of the *dystrophia myotonica protein kinase* (*DMPK*) gene [5]. The severity of the disorder generally increases with the number of CTG repeats: healthy individuals have up to 40 repeats, patients with classic DM1 have 100–1000 repeats, and patients affected by congenital DM1 can have more than 2000 CTG repeats.

A major patho-mechanism underpinning DM1 is the generation of toxic RNAs containing expanded CUG triplets that accumulate as distinctive nuclear foci and dysregulate the activity of RNA processing factors, including MBNL1 and CELF1 as well as Staufen1 and DDX5 [6,7,8,9,10,11,12]. Expanded CUG repeats have been demonstrated to be toxic in several cell types and animal models [13,14,15], disrupting pre-mRNA alternative splicing [16]. RNA splicing alterations result in the re-emergence of developmentally immature alternative splicing and polyadenylation patterns in adult muscles, as well as in alterations of the localization and turnover of specific transcripts [7,16,17,18,19].

Circular RNAs (circRNAs) are covalently closed-loop structure RNAs. They are generated by splicing events occurring on maturing pre-mRNAs in a different order than their genomic sequence, joining together a donor site with an upstream acceptor site [20,21,22,23,24].

CircRNAs have no accessible 5′ or 3′ ends and are not poly-adenylated, escaping detection by many analytical and bioinformatics tools that are widely used in RNA biology. Indeed, for a long time, circRNAs were dismissed as rare aberrant by-products of the splicing process. More recently, large RNA deep-sequencing projects and the development of bioinformatics tools enabling the analysis of extensive data-sets have allowed the identification of significant proportions of back-splice junction reads associated with circRNAs in virtually all eukaryotic organisms [25,26,27,28,29]. While many circRNAs are likely byproducts of RNA splicing mechanisms, some circRNAs can be even more abundant than their linear counterparts [28] and specific biological functions have been associated with a rapidly increasing number of them [24]. Certain circRNAs contain sequences complementary to the seed of a specific microRNA (miRNA), sequestering it and therefore reducing its bioavailability for target-mRNA inhibition [30]. A prototype of this is CDR1AS/ciRS-7, a circRNA containing about 70 evolutionarily conserved binding sites for miRNA-7 [26,31]. Recently, CDR1AS was also found to regulate the turnover-rate of miR-7 in Cdr1as knock-out mice [32]. Certain circRNAs can regulate the expression of their linear counterparts, reducing the amount of pre-mRNA available for canonical splicing. Moreover, exon-intron circRNAs have also been described which can interact with U1 snRNP and promote transcription of their parental genes [33]. Additionally, circRNAs binding and functionally interacting with RNA-binding proteins have been identified. For instance, circMBL, derived from the *MBL*/*MBNL1* gene, in both *D. melanogaster* and humans, contains MBNL1 binding sites; MBL overexpression induces circMBL generation and this effect is dependent on the MBL binding sites [34]. Finally, a small fraction of circRNAs contains the necessary information to be translated with a cap-independent translation mechanism [27,35].

While it is well established that RNA splicing is aberrant in DM1, whether circRNA levels are dysregulated has not yet been explored. With this study, we provide the first evidence that the levels of specific circRNAs linked to myogenesis are deregulated in skeletal muscle biopsies and in myogenic cell cultures derived from DM1 patients. Due to their resistance to exonucleolytic degradation, circRNAs are generally more stable than linear RNAs, constituting an attractive new class of potential biomarkers [36]. Accordingly, we also show here that circRNAs are detectable in both peripheral blood mononuclear cells (PBMCs) and plasma derived from the blood of DM1 patients.

## 2. Results

### 2.1. Identification of circRNA Expression in DM1 Skeletal Muscle by RNA-Sequencing

Published RNA sequencing (RNAseq) data of ribo-depleted libraries derived from five controls and 25 DM1 tibialis anterior biopsies [37,38] were investigated for circRNA expression. Among the available data-sets, we analyzed only libraries containing at least 76 million reads, thus providing sequence information at a high depth (Table S1). By applying CIRI2 [39,40], an algorithm designed to discover circRNAs, a total of 21.822 unique back-splice sites were identified across all the libraries. Since most of these events were present in few samples only, a stringent abundance filter was used, resulting in ~1.800 back-splice junctions (Table S2). Certain circRNAs display expression levels comparable to or even higher than their linear counterparts, suggesting a potential biological relevance [28]. To identify these particularly interesting circRNAs, the circular-to-linear ratios were estimated. Hence, all annotated linear junctions involved with either the donor or the acceptor site of the back-splice event were quantified. The linear junction with the highest expression was then compared to the back-splice junction, revealing 578 circRNAs with a circular-to-linear ratio >0.5 in DM1 (Table S3).

To narrow the list of candidates for validation, the ~1.800 identified circRNAs were intersected with a list of 29 circRNAs that have been previously validated in human and mouse myoblasts [27], resulting in 18 common circRNAs (Table S3). Interestingly, most of them displayed a circular-to-linear ratio >0.5.

### 2.2. Validation by qPCR of Differentially Expressed circRNAs in DM1 Skeletal Muscles

Muscle tissue biopsies were harvested from biceps brachii of 30 DM1 and 29 sex- and age-matched control individuals with no sign of neuromuscular disorders. The DM1 group showed the main characteristics of the disease, including myotonia, cataract, and muscle weakness (Table 1), as well as the typical histological alterations, such as central nuclei, high variation in fiber size, atrophic fibers, and nuclear clumps (Figure S1) [1,41]. Most DM1 patients were at stage 3–4 and the pathological expansions of the CTG triplets ranged from 90 to 1100.

Total RNAs were isolated and the expression of a set of circRNAs and their linear counterparts was measured by qPCR. Out of the 18 circRNA candidates, eight primer couples passed all technical checks of specificity and efficiency. Of note, two circRNAs originated from the same gene and were labelled circRTN4 and circRTN4_03. The primers designed for circRNAs produced an amplicon spanning the back-splice junction, while the linear primers resulted in amplicons crossing the linear junction to a neighboring exon. Due to the key role of MBNL in DM1, the previously identified circRNA hosted in the second exon of *MBNL* [34] and its linear form were also measured. Thus, a set of nine circRNAs together with their linear counterparts were used for validation (Table S4). All tested transcripts were confirmed to also be readily expressed in biceps brachii biopsies, with the only exception being circMBNL1 (circMBNL1), which showed an expression close to the detection threshold. Five circRNAs, namely, circASPH, circCDYL, circHIPK3, circRTN4_03, and circZNF609, displayed a statistically significant increase following multiple comparison testing (*q* < 0.01). For these DM1-deregulated circRNAs (DM1-circRNAs), validation was extended to an additional 10 DM1 and 10 control subjects (Figure S2).

Primer efficiency for all these DM1 circRNAs and their linear counterparts was ≥1.9, with the only exception being linear ASPH, which showed an efficiency of 1.8.

In keeping with the RNA-seq data, the Ct values of linear and circular isoforms displayed similar expression levels (Table S5). The only exception was circular RTN4_03, which showed a >15 times lower mean than linear RTN4_03 and a Ct difference of almost nine cycles.

Primer specificity was confirmed by melting curves showing single peaks for each of the primers. Furthermore, gel electrophoresis showed a single band of expected size for all circular RNA fragments produced by qPCR (Figure S3).

**Figure 1:**
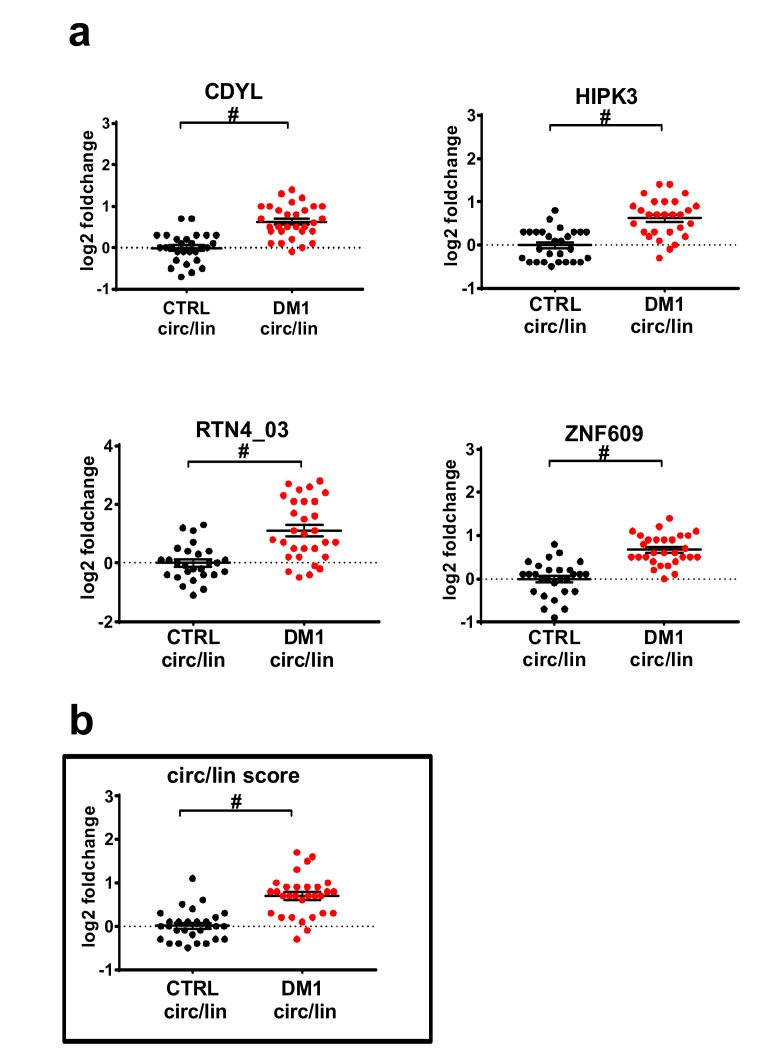
Differentially modulated circular-to-linear ratios in DM1 biceps brachii. (**a**) Scatterplots in log2 scale of significantly different circular-to-linear ratios identified by qPCR in DM1 muscle tissue compared to the control (CTRL). After normality testing, statistical significance was calculated either by a *t*-test or a Mann-Whitney test (threshold *p* < 0.05), followed by correction multiple comparison, with significance threshold set at *q* < 0.01. (**b**) Scatterplot of circular-to-linear ratio score (circ/lin score), estimated by averaging the log2 fold changes of significantly different circular-to-linear ratios in DM1 muscle tissue compared to the control. For both panels, lines indicate mean and standard error values for each group. DM1 = 30 (red dots); CTRL = 29 (black dots); # *q* < 0.00001.

To assess whether the observed induction of the circular transcripts was simply the consequence of a general increase of transcription in the relevant genomic region in DM1 patients, modulation of the ratios between the circular and the linear isoforms was calculated. We identified four circRNAs (circCDYL, circHIPK3, circRTN4_03, and circZNF609) with a significantly increased circular-to-linear ratio in DM1 muscles (Figure 1a), implying a de-regulation of the circular transcript independent from its linear counterpart. Accordingly, a similar trend was also observed in the RNAseq data, where the circular-to-linear ratios were higher in the DM1-affected muscles compared to the controls (Table S3).

As additional validation, published RNA-seq datasets of five controls and nine DM1 quadriceps muscle biopsies [37,38] were analyzed with the stringent criteria described for tibialis anterior. Among the available data-sets, only libraries prepared from high-quality RNAs (RIN ≥ 7) were used. It was found that four DM1-circRNAs were expressed (circASPH, circCDYL, circHIPK3, and circZNF609; Table S6) and displayed high circular-to-linear-ratios (Table S7).

### 2.3. DM1-circRNAs Distinguish DM1 Patients from Controls

**Figure 2:**
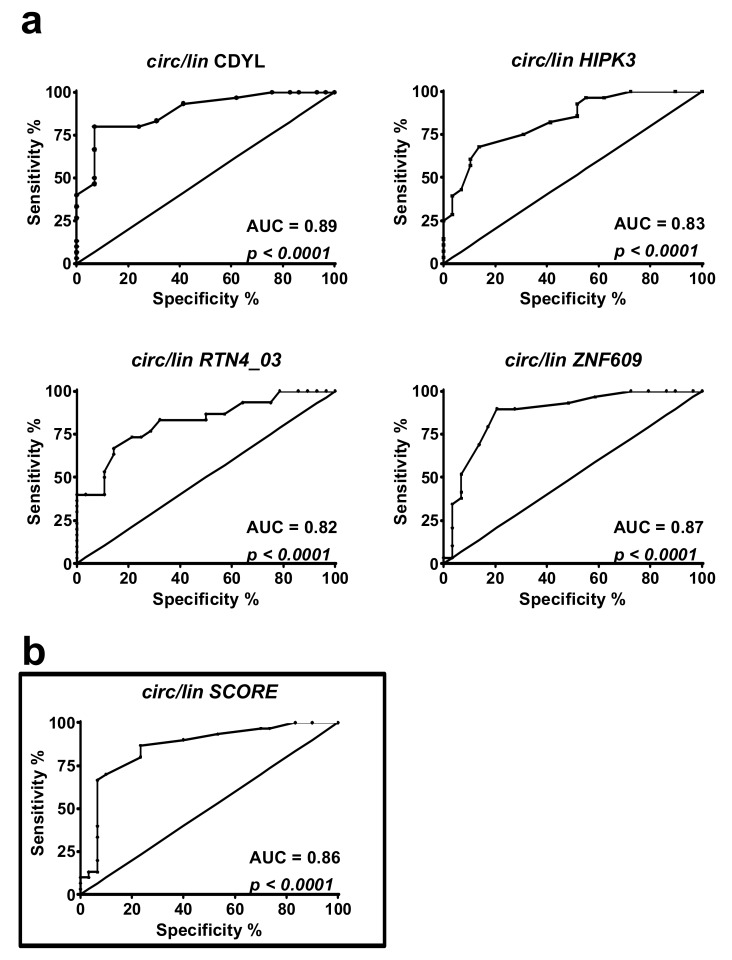
Discrimination of DM1 patients from controls using circular-to-linear ratios of DM1-circRNAs. (**a**) Receiver operating characteristic (ROC) curves show the sensitivity and specificity of each circRNA fraction (circ/lin) and of the combined “circular-to-linear score” to distinguish DM1 from healthy biceps brachii muscle tissue. (**b**) The “circular-to-linear score” was calculated by averaging the significantly modulated circular-to-linear ratios (circ/lin score, black rectangle). DM1 = 30, CTRL = 29.

To understand if the identified DM1-circRNAs displayed the discriminating power to identify DM1 patients, receiver operating characteristic (ROC) curve analysis was performed. Both significantly increased circRNAs alone (Figure S4) and circular-to-linear ratios (Figure 2) were analyzed. In detail, among the tested ratios, CDYL showed the largest area under the curve (AUC) (= 0.89), while the others ranged between 0.82 and 0.87 (Figure 2a). A score was estimated to compensate for the variability of individual DM1-circRNAs, thus improving the signature robustness. Averaging all five DM1-circRNA fractions into a “circular-to-linear score” (Figure 1b) improved the performance (AUC = 0.86) with respect to the singular fractions of HIPK3 and RTN4_03 (Figure 2b).

In conclusion, each of the five circular-to-linear ratios, as well as the combined “circular-to-linear score” of the DM1-circRNAs are useful to discriminate between healthy and diseased patients.

### 2.4. Correlation between DM1-circRNAs and Clinical Characteristics

**Figure 3:**
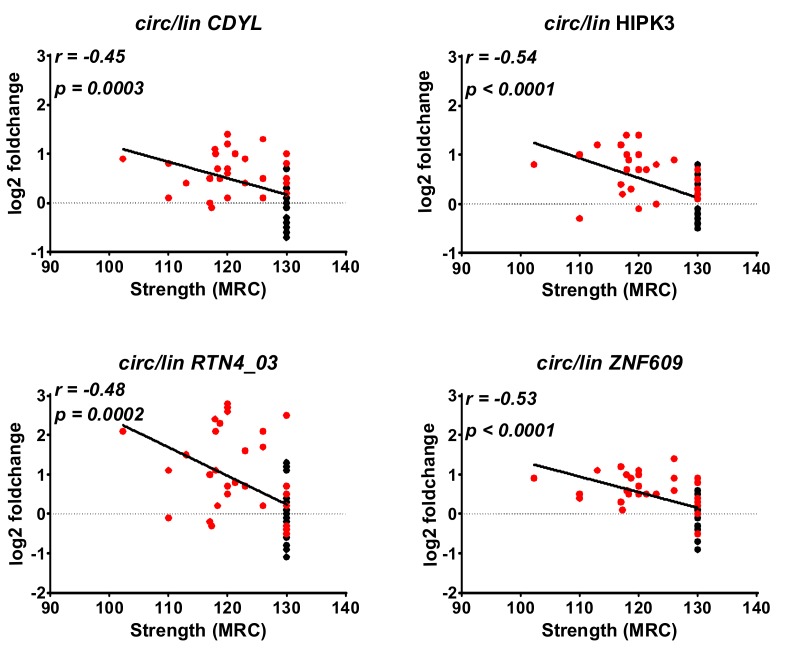
Correlation of muscle strength with circular-to-linear ratios of DM1-circRNAs. Pearson correlation values between significantly modulated circular-to-linear ratios identified in biceps brachii muscle biopsies and muscle strength were measured by Medical Research Council (MRC) megascore. DM1 = 30 (red dots), CTRL = 29 (black dots).

**Figure 4:**
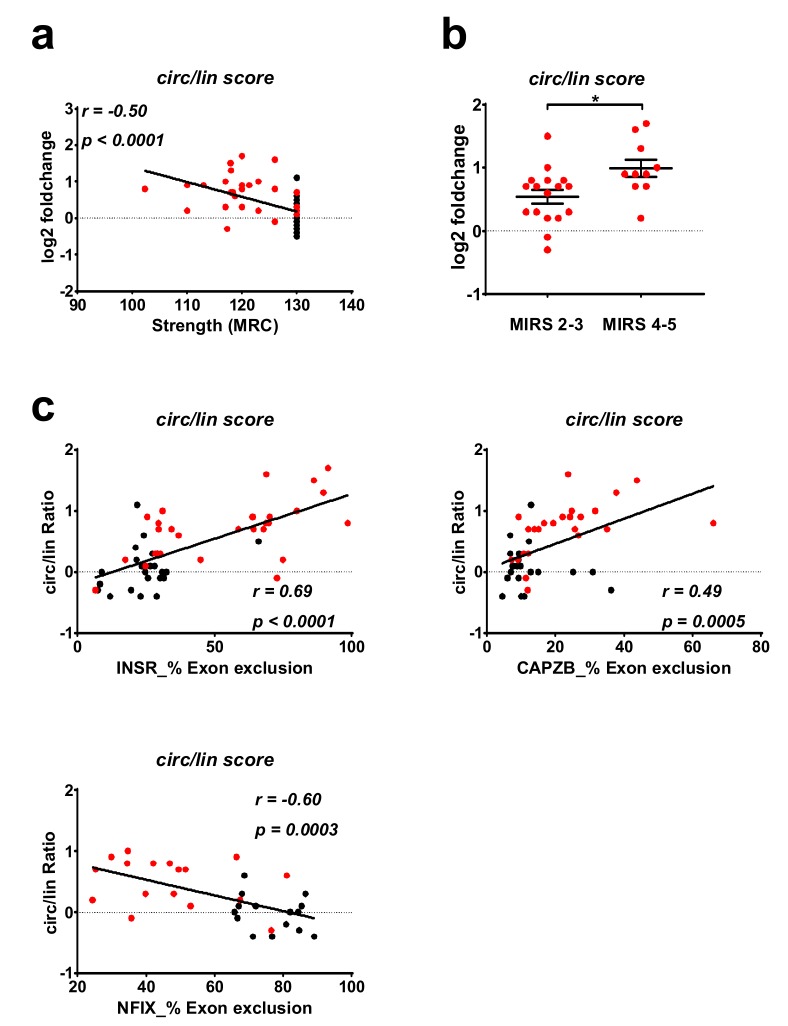
Correlation of DM1-relevant parameters with the circular-to-linear score of DM1-circRNAs. (**a**) Pearson correlation of the circular-to-linear score (obtained by averaging all DM1-circRNA fractions) with muscle strength measured by MRC megascore. (**b**) DM1 patients were divided according to (Muscular Impairment Rating Scale) MIRS classes. *T*-test statistics identified a significant increase (* *p* < 0.05) of the circular-to-linear score in patients belonging to higher MIRS classes (4–5) compared to lower MIRS classes (2–3). (**c**) Pearson correlation of circular-to-linear scores with percentages of exons exclusion of INSR, CAPZB (DM1 = 30, CTRL = 29), and NFIX (DM1 = 17, CTRL = 15). Red dots = DM1, black dots = CTRL.

To evaluate a potential relationship between the deregulation of circRNAs and clinical conditions, correlation analyses were performed. One of the most clinically relevant parameters for DM1 patients is muscle strength, which is measured by the Medical Research Council (MRC) grading system. We found that the changes of the circular fractions of circCDYL, circHIPK3, circRTN4_03, and circZNF609 displayed a moderate but significant negative correlation to MRC (Figure 3). Accordingly, a negative correlation was observed also between the MRC grading and the circular-to-linear score (Figure 4a). The strongest and most significant correlation was found for the circular fraction of HIPK3, with Pearson *r* = 0.54 and *p* < 0.0001. Furthermore, there was a significant increase of circular-to-linear score values in subjects belonging to higher (Muscular Impairment Rating Scale) MIRS stages (4–5), displaying a more severe disease, compared to less severe MIRS stages (2–3) (Figure 4b). Collectively, these data encourage further investigation of the potential of circRNAs as DM1 biomarkers.

### 2.5. Correlation of DM1-circRNA Levels and Alternative Splicing Events

The webresource DM-seq [38] was used to correlate the DM1 splicing signature identified in the same tibialis anterior data sets to the expression levels of DM1-circRNAs to splicing biomarkers of disease severity [42] (Table S8). This analysis revealed low but significant correlations between the normalized counts of circHIPK3, circRTN4_03, circRTN4, circZNF609, and the “percentage spliced in” (PSI) values of INSR and NFIX. Expression of circCDYL correlated significantly to splicing events in NFIX and CAMK2B. Interestingly, the isoform circRTN4 showed significance in correlation to four additional DM1 splicing events in CAMK2B, CACNA1S, CAPZB, and MBNL1. The DM1-score (averaging the expression of validated circRNAs) correlated significantly to DM1 splicing in INSR and NFIX.

Furthermore, DM1 splicing events in INSR, CAPBZ, and NFIX were investigated by PCR in the biceps brachii cohort. Indeed, significant correlation between the circular-to-linear score and the percentage of exon exclusion for all three splicing events could be found (Figure 4c, Table S9). In detail, circular-to-linear ratios for CDYL, HIPK3, ZNF609 showed low to moderate, but significant correlations to all three alternative splicing events. Instead, the circular-to-linear ratio of RTN4_03 reached significance only for the splicing event in INSR and CAPBZ (Table S9).

### 2.6. DM1-circRNA levels in PBMCs and plasma of DM1 patients

Since peripheral blood can be obtained with a minimally invasive procedure, it represents a potentially interesting tissue for biomarker identification. Thus, we measured DM1-circRNA expression in PBMCs and plasma of DM1 patients and sex- and age-matched controls. Diseased and healthy subjects were chosen with the same criteria adopted for the harvesting of skeletal muscle biopsies (Table S10).

In PBMCs all tested DM1-circRNAs were readily detectable, but none of them showed a significant modulation (Figure S5). In plasma samples only circCDYL and circRTN4 were readily detectable. For both, circCDYL and circRTN4, a small but not significant induction in DM1 patients could be observed. This trend is in line with the data obtained in biopsies (Figure S6).

### 2.7. DM1-circRNA Expression in DM1 Myogenic Cell Lines

**Figure 5:**
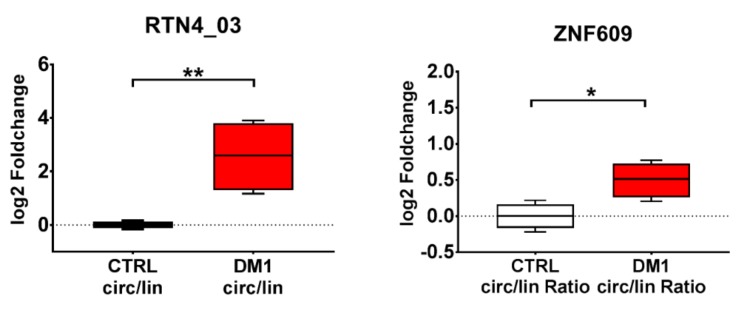
Differentially modulated circular-to-linear ratios in myogenic cell lines. Boxplots of significantly different circular-to-linear ratios identified by qPCR in differentiated DM1 myogenic cells compared to controls (* *p* < 0.05; ** *p* < 0.01; DM1 = 4; CTRL = 4).

We assessed whether circRNA alterations identified in DM1 muscle biopsies were also observed in cultured myoblasts. To this aim, we took advantage of DM1 and control muscle cell lines obtained by conversion of immortalized skin fibroblasts into multinucleated myotubes by forced expression of *MyoD1* [43]. In DM1 and control differentiated myogenic cells, all circRNAs tested were readily detectable. Interestingly, two of the circular transcripts, circRTN4 and circRTN4_03, were significantly increased in DM1 compared to the control (Figure S7). The analysis of their circular-to-linear ratio confirmed the induction only for the isoform RTN4_03. Additionally, while the circular transcript of ZNF609 alone did not reach significance, its circular-to-linear ratio was significantly increased in DM1 compared to the control subjects. These results are in line with the findings obtained in DM1 biopsies (Figure 5).

We took advantage of this cell culture system to investigate whether silencing of the DM1-related splicing factor affected the levels of the DM1-circRNAs modulated in vitro.

Since MBNL1 is impaired in DM1 patients [7,9,10], we assayed whether MBNL1 silencing in control myogenic cells induced, at least in part, the circRNA deregulations observed in DM1 myogenic cells. Control differentiated myogenic cells were transfected with MBNL1 siRNAs or relevant control oligonucleotides and RNA was extracted three days later. MBNL1 mRNA was significantly down modulated (Figure S8a) and the expected alterations in the alternative-splicing patterns of SERCA1 and insulin receptor (IR) were observed (Figure S9b,c) [44,45]. However, no increase was observed in the abundance of circZNF609, circRTN4, and circRTN4_03 levels (Figure S8d).

We also tested whether the silencing of CELF1, which is activated in patients and in DM1 disease models [46], rescued DM1-circRNA expression in DM1 differentiated myogenic cells. In spite of effective CELF1 knock-down (Figure S9a), no significant change of circZNF609, circRTN4, and circRTN4_03 levels was observed (Figure S9b).

We conclude that the DM1 myogenic cell lines studied reflect, at least in part, the outcome of the DM1 biopsies and therefore represent a valuable tool for functional studies in vitro.

## 3. Discussion

A molecular hallmark of DM1 is the dysregulation of alternative splicing, which affects many genes involved in muscle homeostasis and function [16,17,42]. Since circRNA biogenesis competes with canonical splicing during pre-mRNA maturation, this emerging class of non-coding RNAs can be regarded as alternative splicing products. Via a so-called back-splice event they form a single stranded, covalently closed RNA transcript. [20,21]. In this pilot study, we provide evidence of de-regulation of circRNA expression in DM1 patients. We analyzed 30 publicly available gene-expression data-sets of DM1 and control tibialis anterior muscles [37,38] using bioinformatics tools designed for the identification of circRNA-specific back-splice events. Applying stringent selection and abundance filters, we identified ~1.800 unique circular splicing events, a number comparable to circRNAs found to be expressed in human myoblasts and myotubes [27].

Due to very low read numbers for most circRNA species, the identification of circRNAs potentially relevant in DM1 was not guided by differential expression analysis. Instead, we filtered for circRNAs displaying expression across many samples and then selected circRNAs previously shown to be involved in myogenesis [27]. Of note, most of these circRNAs also displayed a high circular-to-linear ratio. This suggests that these circRNAs are not a mere by-product of the transcript maturation process, and might also indicate an independent regulation as well as an additional biological function.

For opportunity-related reasons, the validation step was performed in biceps brachii biopsies, since only for this muscle type a sufficient number of samples was available to us. It should be acknowledged that biceps brachii is generally less severely affected than other distal muscles in DM1 patients [1,2,3,41]. Thus, some of the circRNA level differences that failed to reach statistical significance in our validation analysis might be indeed relevant in distal muscles. On the other hand, it is plausible to hypothesize that circRNA alterations identified in proximal muscles could be more pronounced in distal muscles which are affected more severely.

In spite of the not-comprehensive nature of this study, we found that the levels of five out of nine circRNAs tested were significantly increased, suggesting a potentially pervasive dysregulation of circRNAs in DM1. Accordingly, four of these circRNAs also displayed an increased circular-to-linear ratio. These results were confirmed by the analysis of publicly available gene-expression data-sets of DM1 and control quadriceps muscles [38]. The most likely interpretation of these alterations is that they are caused by the dysfunction of the alternative splicing machinery characterizing DM1 [16,17,42]. Silencing of either MBNL1 or CELF1 in the control and DM1 cultured myotubes did not affect the abundance of circZNF609, circRTN4, and circRTN4_03. While negative data should always be evaluated in a very cautious manner, one possible interpretation is that other splicing factors, such as different MBNL-family members, e.g., Staufen1 and DDX5 [6,7,8,9,10,11,12], regulate the generation of the DM1-circRNAs. Moreover, these splicing factors might be redundant in circRNA regulation, making the silencing of a single splicing factor ineffective. Finally, higher stability of circRNAs compared to their linear counterparts [47] should also be considered as a possible mechanism underpinning the increase of the circular-to-linear ratio of DM1-circRNAs.

CircRNA dysregulation may lead to pathological consequences to be investigated [24]. In this respect, however, little is known of the identified DM1-circRNAs. CircHIPK3 positively regulates human cell growth by sponging multiple miRNAs [48,49]. Among these miRNAs are miR-29b and miR-193a. Intriguingly, both miRNAs were previously found to be down-modulated in DM1 [50] and DM2, respectively [51]. Moreover, circHIPK3 can act as a sponge of miR-30a, inhibiting its anti-miogenic function and promoting the proliferation and differentiation of chicken myoblasts [52]. CircHIPK3 levels are also increased in retinal endothelial cells exposed to diabetes-related stressors in retinas of diabetic mice and in the plasma of diabetic patients. Moreover, in retinal endothelial-cells, circHIPK3 affects cell viability, proliferation, migration, and function [53]. While the implications of circHIPK3 in DM1 should be investigated, it is worth noting that insulin resistance is very often present in DM1 patients [1,2,3,54].

CircZNF609 is downregulated during myogenesis and can specifically control myoblast proliferation [27]. Moreover, its mouse homologue circZfp609 suppresses myogenic differentiation [55]. Of note, circZNF609 is elevated in Duchenne muscular dystrophy myoblasts, indicating that the DM1-circRNAs identified in this study might be deregulated in other muscular diseases. Like circHIPK3, circZNF609 is also induced by high glucose in vivo and in vitro and regulates the function of retinal endothelial cells [56].

Finally, the host gene of circRTN4 displays muscle-specific splicing [57]. It is a direct target of RBM20, an alternative-splicing regulator of cardiac genes which is associated with coronary heart disease. Mutation of *RBM20* results in altered splicing of its target genes, causing the retention of specific exons of RTN4 mRNA. Since RBM20 is also expressed and active in skeletal muscles [58], further investigations are needed to also assess the potential involvement of RBM20 in circRTN4 formation.

In neuronal tissues of flies and humans, the second exon of the splicing factor *MBLN1* (*MBL* in drosophila) is circularized, competing with canonical pre-mRNA splicing. Moreover, Muscleblind protein interacts with flanking introns of its own gene to promote exon circularization [34]. This observation prompted us to measure circMBNL1 in skeletal muscle biopsies and PBMCs of DM1 patients, where MBNL1 protein bioavailability is reduced by its sequestration in nuclear CUG-foci [9,10]. However, we did not observe any modulation of circMBNL1 in these tissues. The most likely explanation is that circMBNL1 regulation is highly context specific. Indeed, while in fly heads, this circRNA is more abundant than the linear counterpart, the opposite seems to be true in human skeletal muscle, where RNAseq data indicated a circular-to-linear ratio of 0.05 (Table S3). In keeping with this hypothesis, very low circMBL levels were also observed in drosophila S2 cells [34]. Furthermore, changes in the splicing pattern of MBNL1 mRNA (comprising or not exon1) were observed in cardiac and skeletal muscles, but not in the brain of DM1 patients compared to controls [59,60], confirming a high tissue-specificity in the regulation of MBNL1 transcript.

Identification of diagnostic and prognostic biomarkers is an unmet clinical need for DM1 patients. Recent studies suggest that alternative splicing isoforms in skeletal muscle tissue have high potential as biomarkers of DM severity and for the monitoring of therapeutic responses [42]. Due to their loop-structure, circRNAs are highly resistant to exonucleases [47], holding great potential as disease biomarkers. Promisingly, the potential of circRNAs as molecular markers has been highlighted recently in various types of cancer [61], measuring circRNAs not only in biopsies of solid tissues but also in extracellular compartments, such as serum or exosomes [61,62].

It is still too early to conclude whether circRNAs will find their way to the clinic. This will largely depend on both technical issues, such as the detectability and stability of circRNAs in biological samples, as well as on whether circRNAs are functional elements of the molecular mechanisms driving the disease or mere byproducts [47]. While further studies are obviously needed, we found that the circular-to-linear ratios as well as the combined “circular-to-linear score” of the DM1-circRNAs in skeletal muscle biopsies accurately discriminated healthy from DM1 patients. Moreover, a moderate correlation between the MRC grading and the circular-to-linear score could be identified. Interestingly, the circular-to-linear score was also found to be increased in patients with a higher MIRS score, potentially reflecting disease severity. Finally, DM1-circRNAs circular-to-linear ratios correlated with the levels of alternative splicing isoforms of INSR, CAPZB, and NFIX, which display good potential as biomarkers of DM severity and therapeutic response [42].

Additional investigations are needed to evaluate the possible potential of circRNAs as DM1 biomarkers which involve a higher number of subjects. Comparison with other myopathies will also allow for the investigation of their possible disease-specificity. Indeed, alteration of the alternative splicing machinery is present in other myopathies and diseases as well, and the observed changes might be secondary events of the DM1 phenotype [63].

CircRNAs are also detectable in peripheral blood that can be harvested with minimally-invasive techniques. Indeed, DM1-circRNAs were measured both in PBMCs and, at least in part, in plasma samples. On the one hand, the highly context-dependent regulation of circRNAs implies that the pattern of expression observed in peripheral blood is unlikely to mirror that of other tissues, such as skeletal muscle. On the other hand, the apparent pervasiveness of circRNA dysregulation suggests that comprehensive screenings of circRNAs might indeed succeed in the identification of common circRNAs between blood and affected organs. An alternative approach may be represented by the analysis of urine extracellular RNAs, whose splice variants have been shown to discriminate DM1 patients efficiently [64].

## 4. Materials and Methods

### 4.1. RNAseq and Bioinformatics Analysis

Taking advantage of the GEO (www.ncbi.nlm.nih.gov/geo/) and DMseq (http://dmseq.org/) repositories, we analyzed RNAseq data-sets derived from tibialis anterior muscle biopsies taken from DM1 and control patients (GSE86356) [37,38]. In order to ensure sufficient sequencing depth for the identification of the expectedly rare back-splicing events, we used only data-sets containing more than 75 million reads (Table S1). In this way, raw reads in fastq format from five control and 25 DM1 ribo-depleted libraries were aligned to a hg19 reference genome with BWA software (version 0.7.12, Wellcome Trust Sanger Institute, Wellcome Trust Genome Campus, Cambridge, UK), with options chosen according to a CIRI2 manual [39,40].

Subsequently, circRNAs were identified by detecting back-splice events in each of the aligned samples using CIRI2 (version 2.0.6, Computational Genomics Lab, Beijing Institutes of Life Science, Chinese Academy of Sciences, Beijing, China) with the suggested parameters. All identified circRNAs were then collected, normalized to each library size, and quantified using custom R scripts. An abundance filter was applied by removing back-splice events present in <70% of either the control or DM1 samples (Table S2). The circular-to-linear ratio was calculated using the highest expressed linear junction involved in the relevant back-splice event (Table S3). Briefly, we compared all annotated linear junctions that comprised either the acceptor or the donor site of the back-splice junction and kept that with the highest amount of spliced reads. This ensured that the ratio was determined by using two equal biological entities, i.e., reads spanning a splice junction.

Additionally, RNAseq data obtained from quadriceps muscle biopsies included in the same study (GSE86356) [37,38] were used for circRNA identification. Among the available data sets, only libraries prepared with high-quality RNA were used (RIN ≥ 7). Identification, quantification of circRNAs, and estimation of circular-to-linear ratios were performed as described above.

Data on alternative splicing events in DM1 available on the DM-seq platform [38] were downloaded for the same tibialis anterior data sets used for circRNA analysis. In detail, PSI values determined by MISO software (hg19 v2.0 MISO version, Airoldi and Burge, Cambridge, MA, USA) of a subset of 10 splicing biomarkers of disease severity in DM1 were correlated to circular-to-linear ratios of DM1-circRNAs.

### 4.2. Patient Characteristics and Tissue Collection

Clinical diagnosis of DM patients was based upon the criteria set by the International Consortium for Myotonic Dystrophies guidelines [41]. Genetic analysis was carried out to confirm DM1 diagnosis as described previously [65]. MIRS was used to determine the disease stage [66]. The MRC scale was used to evaluate muscle strength.

Biceps brachii muscle biopsies collected from 30 DM1 patients and 29 sex- and age-matched subjects without signs of neuromuscular disorders (controls) were used for validation (Table 1).

PBMCs were isolated from the peripheral blood of 19 DM1 and 18 sex- and age-matched controls (Table S6) by Ficoll-Paque™ PLUS (Ge Healthcare, Chicago, IL, USA) gradient centrifugation as described before [67]. The plasma of 29 DM1 and 28 age-and sex- matched controls (Table S6) was collected in EDTA-tubes and cells, and platelets were removed as described previously [68,69].

### 4.3. Ethical Approval and Informed Consent

The experimental protocol was reviewed and approved by the Institutional Ethics Committee of the San Raffaele Hospital (protocol number: miRNADM, of 23 June 2015, Ethics Committee of San Raffaele Hospital) and was conducted according to the principles expressed in the Declaration of Helsinki, institutional regulation, and Italian laws and guidelines. Written informed consent was obtained from each patient prior to muscle biopsies or blood collection.

### 4.4. Histopathological Analysis

Muscle tissue was fresh-frozen in isopentane cooled in liquid nitrogen. Histopathological analyses were performed on serial sections (8 µm) processed for routine histological or histochemical stainings. Myofibrillar ATPase staining was performed as previously described following sample pre-incubation at pH 4.3, 4.6, and 10.4 [70].

### 4.5. DM1 Myogenic Cell Lines

Immortalized human myotonic dystrophy muscle cell lines expressing murine *Myod1* cDNA under the control of a Tet-on inducible construct have been previously described [43]. Cells were cultivated in DMEM growth medium (15% FBS) until confluency was reached. Myogenic differentiation was induced by switching cell cultures to DMEM supplemented with 5 µg/mL insulin and 4 µg/mL doxycycline (Sigma-Aldrich, St. Louis, MO, USA).

For silencing experiments, cells were transfected after three days of differentiation with 50 nM MBNL1/CELF1 TARGETplus SMARTpool siRNAs (Dharmacon, Lafayette, CO, USA ) or with 50 nM ON-TARGETplus Non-targeting Pool as a negative control. Cells were transfected using HiPerFect reagent (Qiagen, Hilden, Germany), according to the manufacturer’s instructions. Three days after transfection, total RNA was isolated and analyzed by qPCR.

### 4.6. Isolation of Total RNA

Total RNA was extracted from muscle tissues using TRIzol reagent (Thermo Fisher Scientific Inc., Waltham, MA, USA) as described previously [51,71]. For isolation of total RNA from PBMCs and cells, TRIzol reagent was used according to the manufacturer’s instructions. The purity and concentration of the obtained RNAs was measured by Nanodrop (Thermo Fisher Scientific Inc.).

Total RNA from plasma samples was extracted as previously described using NucleoSpin miRNA Plasma columns (Macherey-Nagel, Düren, Germany) [68,69].

### 4.7. Real-Time Reverse Transcriptase qPCR

For validation experiments, total RNA was first retro-transcribed using the SuperScript Reverse Transcriptase kit (Version III or IV) and then investigated by SYBR green qPCR according to the manufacturer’s protocol (Thermo Fisher Scientific Inc.). Primer couples were designed by Primer-BLAST tool (Table S4). The circRNAs primers spanned the back-splice junction, while the primer couples for the linear transcripts crossed the linear junction to a neighboring exon. Primer efficiency analysis was performed for all DM1-circRNAs together with their linear counterparts. Additionally, the specificity of primers was assessed by performing melting curve analysis and verification of expected amplicon size by agarose gel electrophoresis.

The relative expression was calculated using the comparative Ct method 2^−∆∆CT^ [72], normalizing to the averaged Cts of RPL13, RPL23, and UBC for tissue RNAs and to the averaged Cts of miR-106a and miR-17-5p for plasma RNAs [68,69].

The circular-to-linear ratio was estimated by subtracting the raw Ct of the linear transcript from the raw Ct of the corresponding circular transcript.

For score-calculations, the log2 fold changes of all significantly modulated circRNAs or circular-to-linear ratios were averaged.

### 4.8. Quantification of Alterntative Exon Usage

RT-PCR for differentially spliced exons in INSR (exon 11 exclusion), CAPZB (exon 8 exclusion), and NFIX (exon 11 inclusion) was performed using Platinum Taq Polymerase (Invitrogen, Carlsbad, CA, USA) using the primers indicated in Table S4. The amplified products were separated on EtBr-stained agarose gels (Sigma-Aldrich) and images were captured using Odyssey^®^ FC (LI-COR). The quantitative analysis of each band was performed by Image Studio Lite 5.2 software and the percentage of exon exclusion calculated.

### 4.9. Statistical Analysis

GraphPad Prism 7.01 (GraphPad Software Inc., San Diego, CA, USA) was used for statistical analysis and for graph generation. Following differential expression analysis, all data-sets were checked for their distribution by D’Agostino and Pearson normality testing. The only exception was the cell line data, since due to the small sample size normality testing was not possible. The differential expression of circRNAs in biopsies was investigated by a multiple *t*-test using the recommended settings of GraphPad for a false discovery rate (Benjamini, Krieger and Yekutieli) and *Q* = 1% as the significance cut-off. For differential expression analysis of circRNAs in PBMCs, plasma, and cell lines, a two-tailed student’s *t*-test or Mann Whitney was used, depending on the data-distribution. A *p* < 0.05 value was deemed statistically significant. Values have been expressed as ± standard error.

## Figures and Tables

**Table 1:** Clinical data on myotonic dystrophy type 1 (DM1) and control patients used for validation in biceps brachii biopsies. Legend: N.R., not relevant; N.A., not available. CTRL, controls; S.E., standard error; MRC, Medical Research Counsil; CK, creatine kinase; ECG-QRS, complex of Q, R and S waves in the electrocardiogram.

Clinical Characteristics	DM1 (*n* = 30)	CTRL (*n* = 29)
Age at sampling (average ± S.E.)	42.2 ± 2.5	41.1 ± 1.1
Sex (male/female)	15/15	20/9
MRC megascore (average ± S.E.)	121.1 ± 1.3	130 ± 0.0
Myotonia (% of patients)	80	0
Glucose (normal values: 70–110 mg/dL)	88.4 ± 4.3	89.0 ± 3.8
Cholesterol (normal values: <200 mg/dL)	212.1 ± 10.4	230.8 ± 24.3
CK (normal values: male <190 mg/dL; female <125 mg/dL)	Male: 266.8 ± 36.4	N.A.
	Female: 221.9 ± 40.2	N.A.
Arrhythmia (% of patients)	17.6	0
Cataract (% of patients)	17.6	0
ECG-QRS duration (normal values: 60–110 ms)	105.2 ± 6.7	N.A.
Number of CTG repeats (range)	494.1 ± 43.9 (90–1100)	N.A.
Stage of disease (range 1–5) (% of patients at each stage)	Stage 1:0	N.R.
	Stage 2:25.9	
	Stage 3:37.0	
	Stage 4:33.3	
	Stage 5:3.7	

## Supplementary Materials

Supplementary materials can be found at https://www.mdpi.com/1422-0067/20/8/1938/s1.
