# Supplementary material for: Dysregulation of Circular RNAs in Myotonic Dystrophy Type 1
Source: Int J Mol Sci. 2019 Apr 19;20(8):1938. doi: 10.3390/ijms20081938 (PMC6515344; doi:10.3390/ijms20081938)
Supplement: Supplementary file 1 [file ijms-20-01938-s001.zip › Supplementary/IJMS_sSupplementary_Figures.S1-9.pdf]

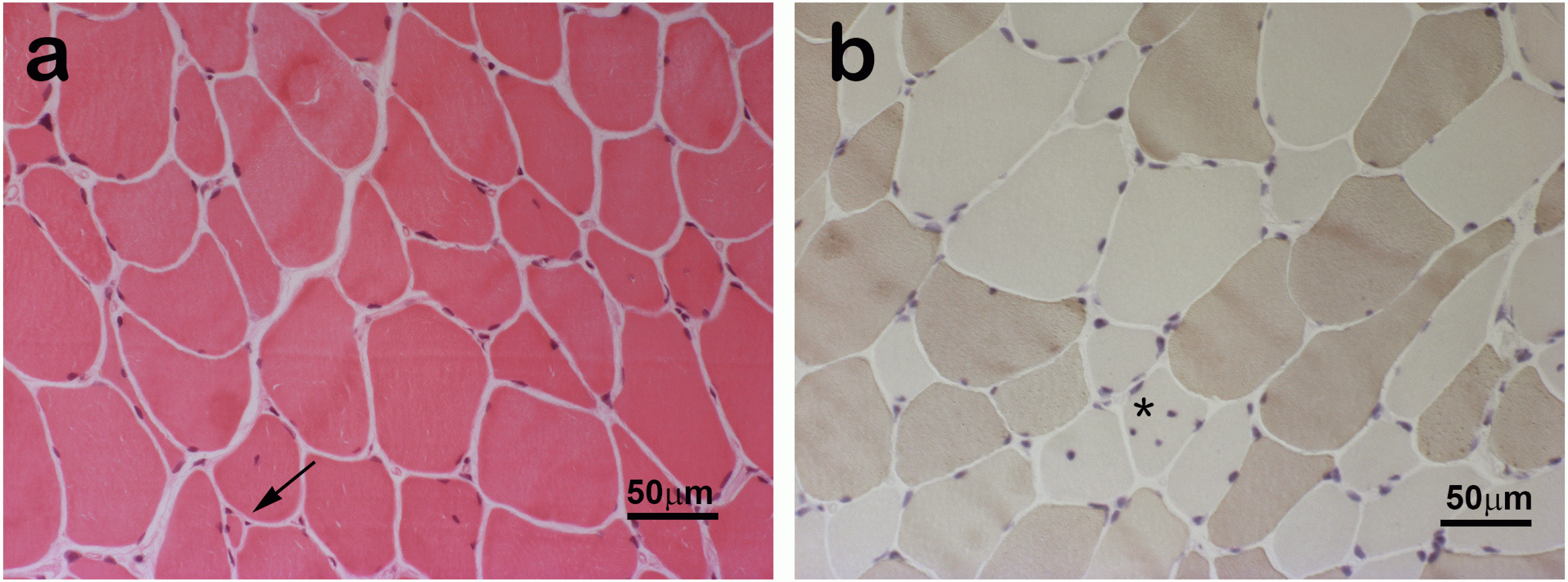


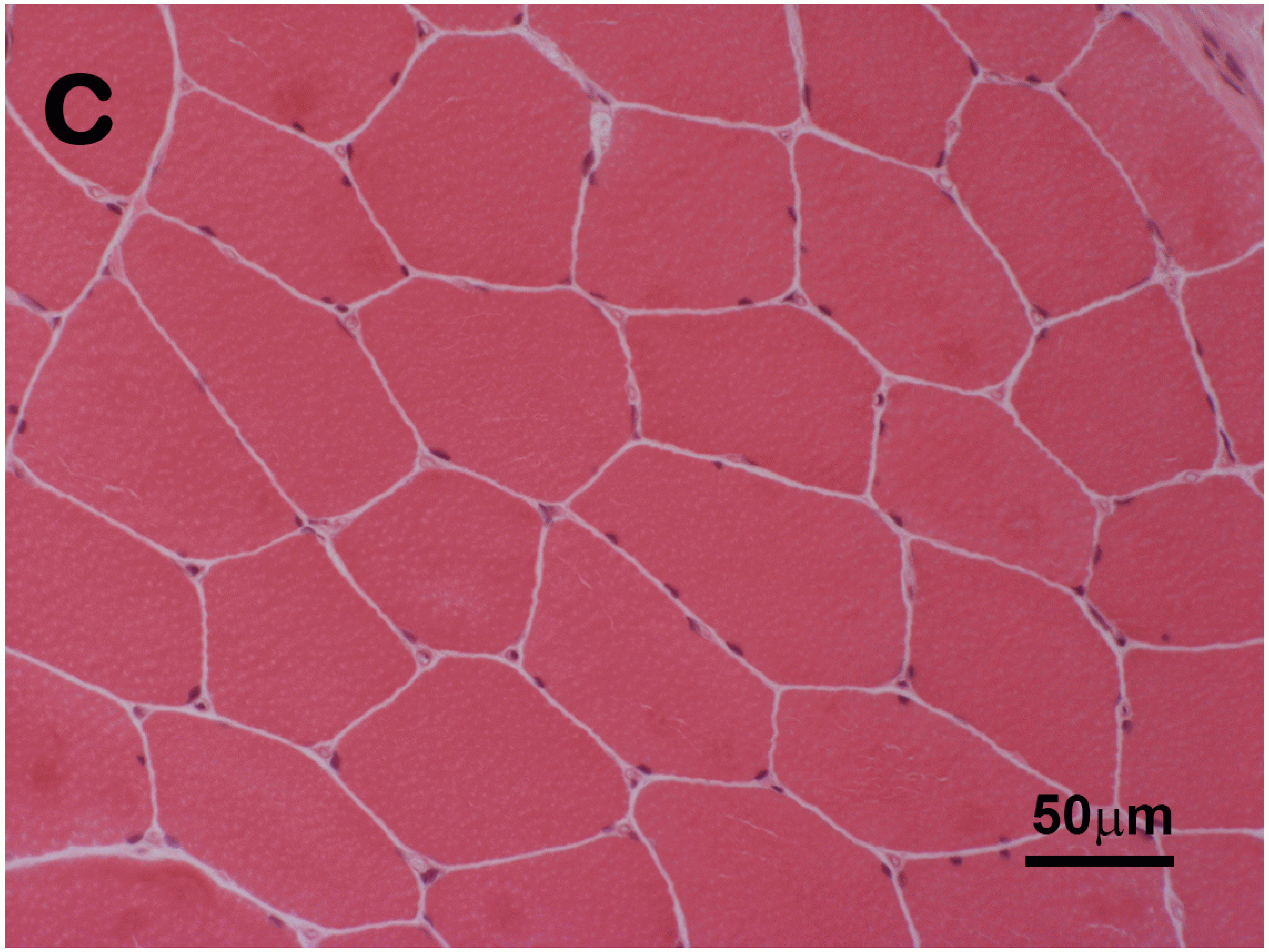


**Supplementary_Figure S1**

# Supplementary Figure S2

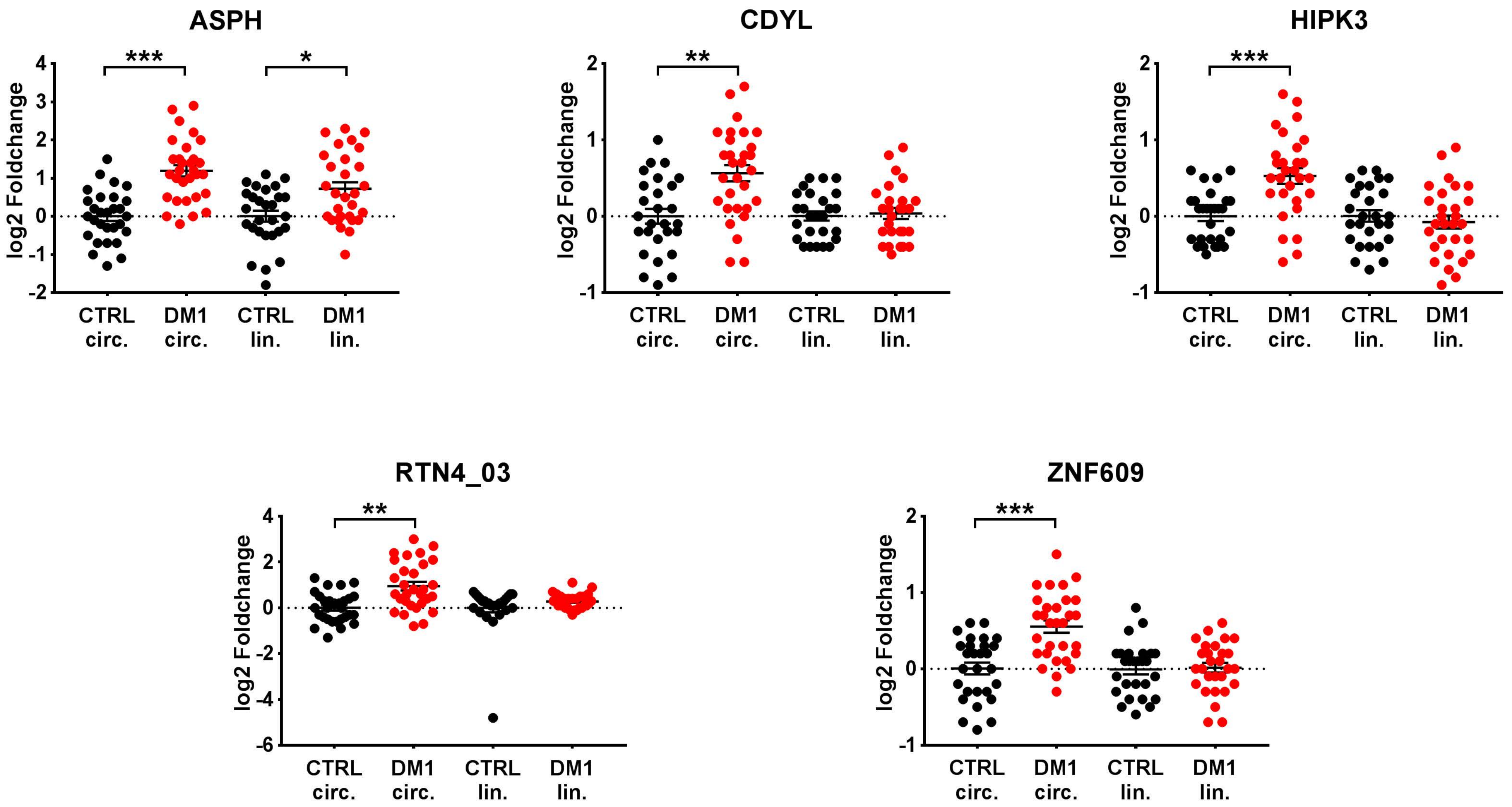

**a**

# Supplementary Figure S3

## CIRCULAR TRANSCRIPTS

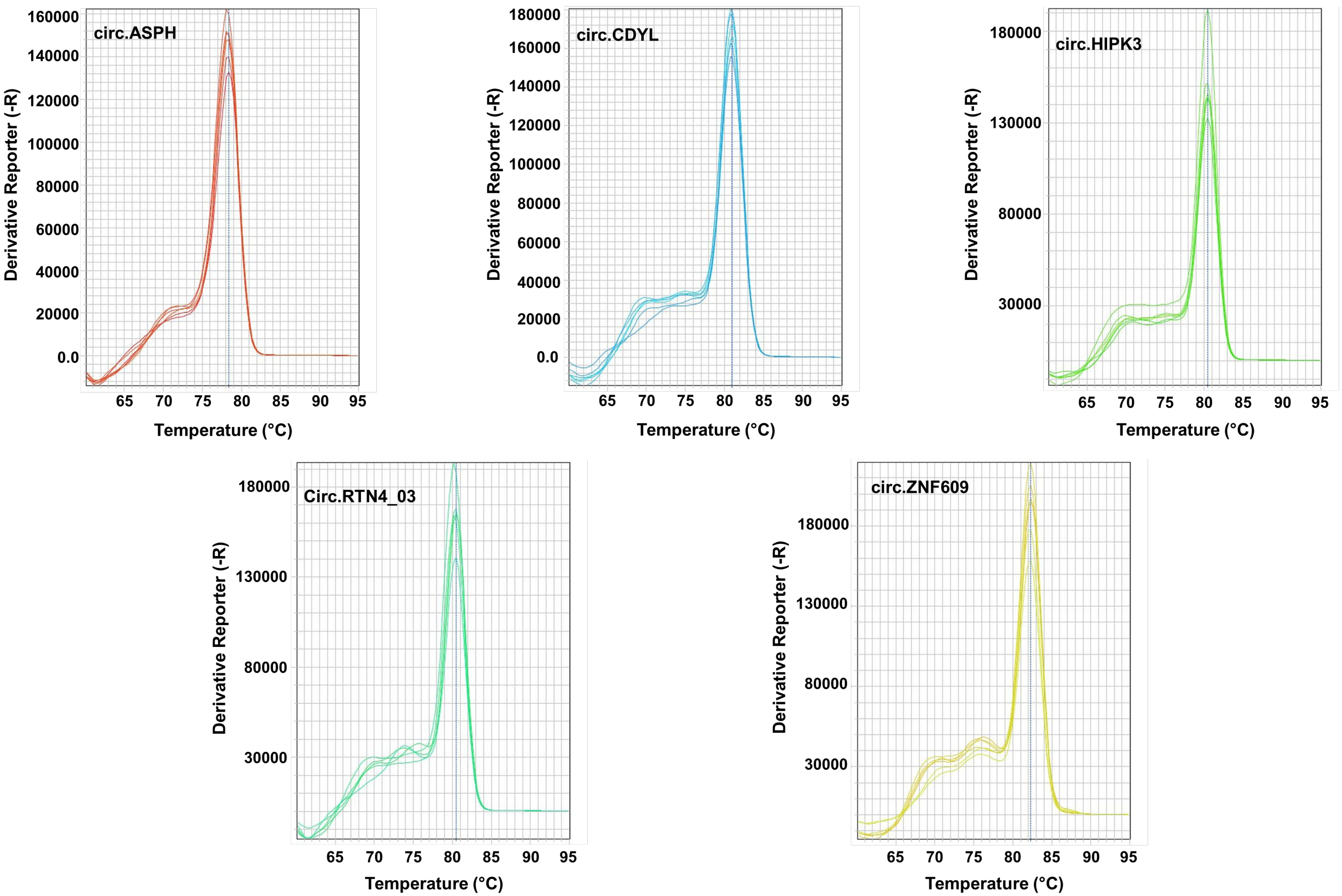

b

# LINEAR TRANSCRIPTS

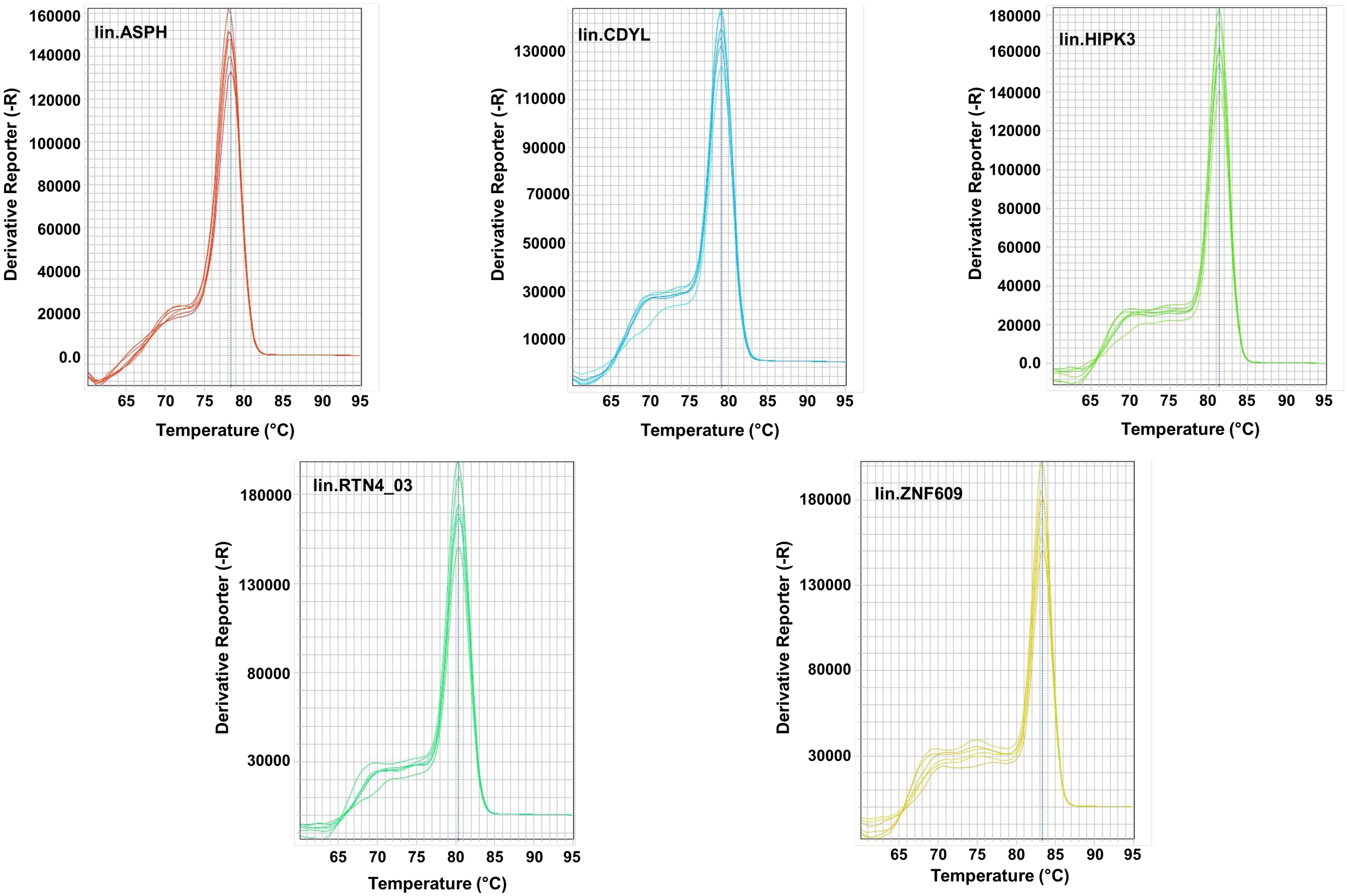

**c**

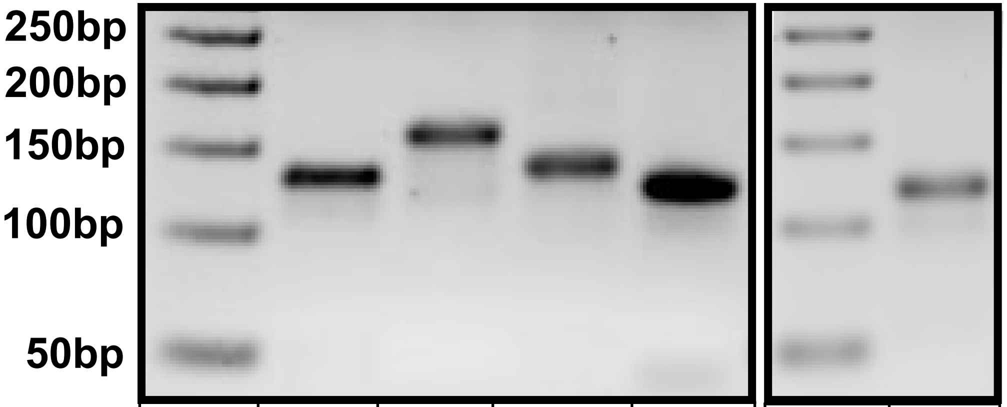


| marker | CDYL | HIPK3 | RTN4_03 | ZNF609 | marker | ASPH |
|---|---|---|---|---|---|---|
| bp | 130 | 155 | 136 | 125 | bp | 120 |

# Supplementary Figure S4

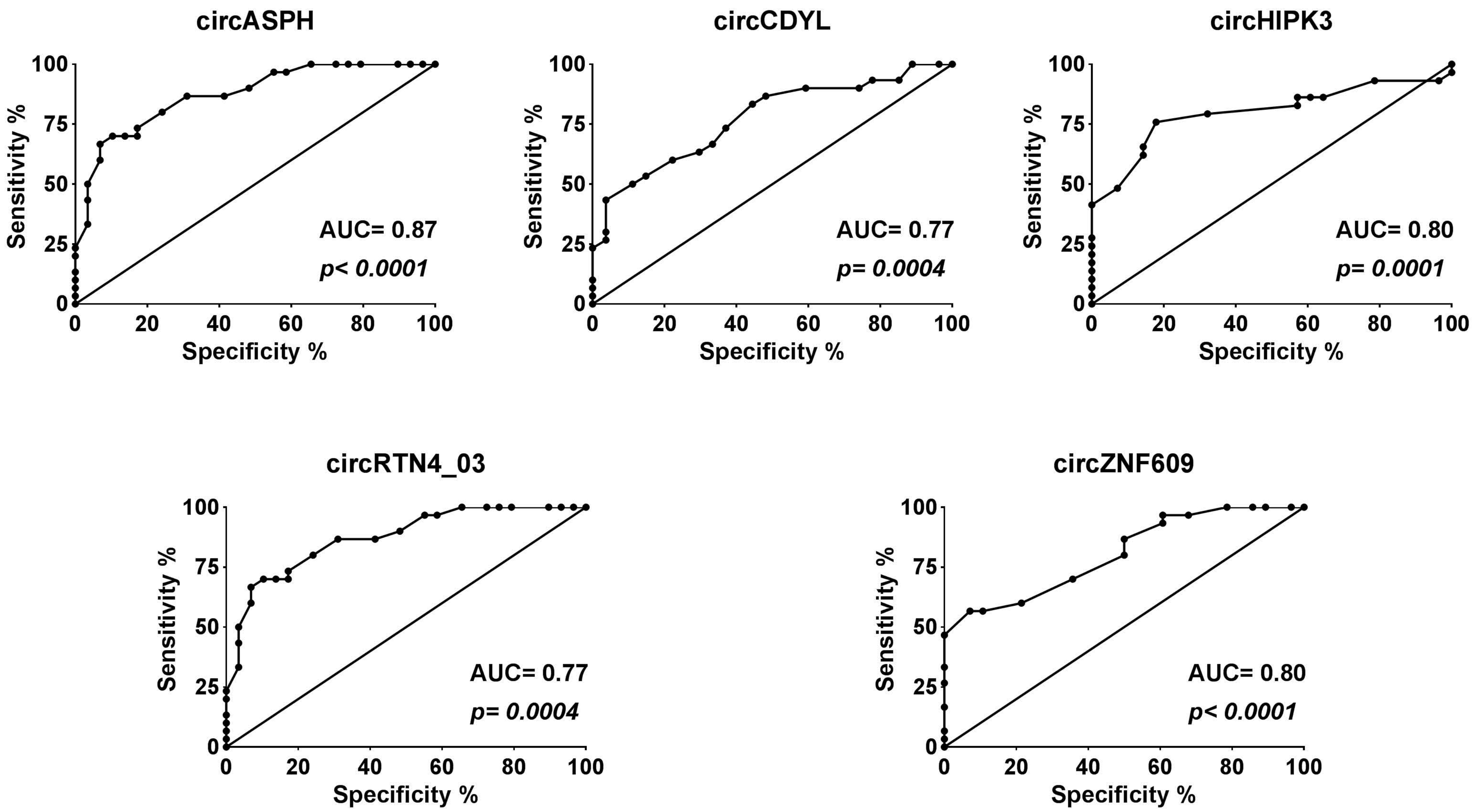

# Supplementary Figure S5

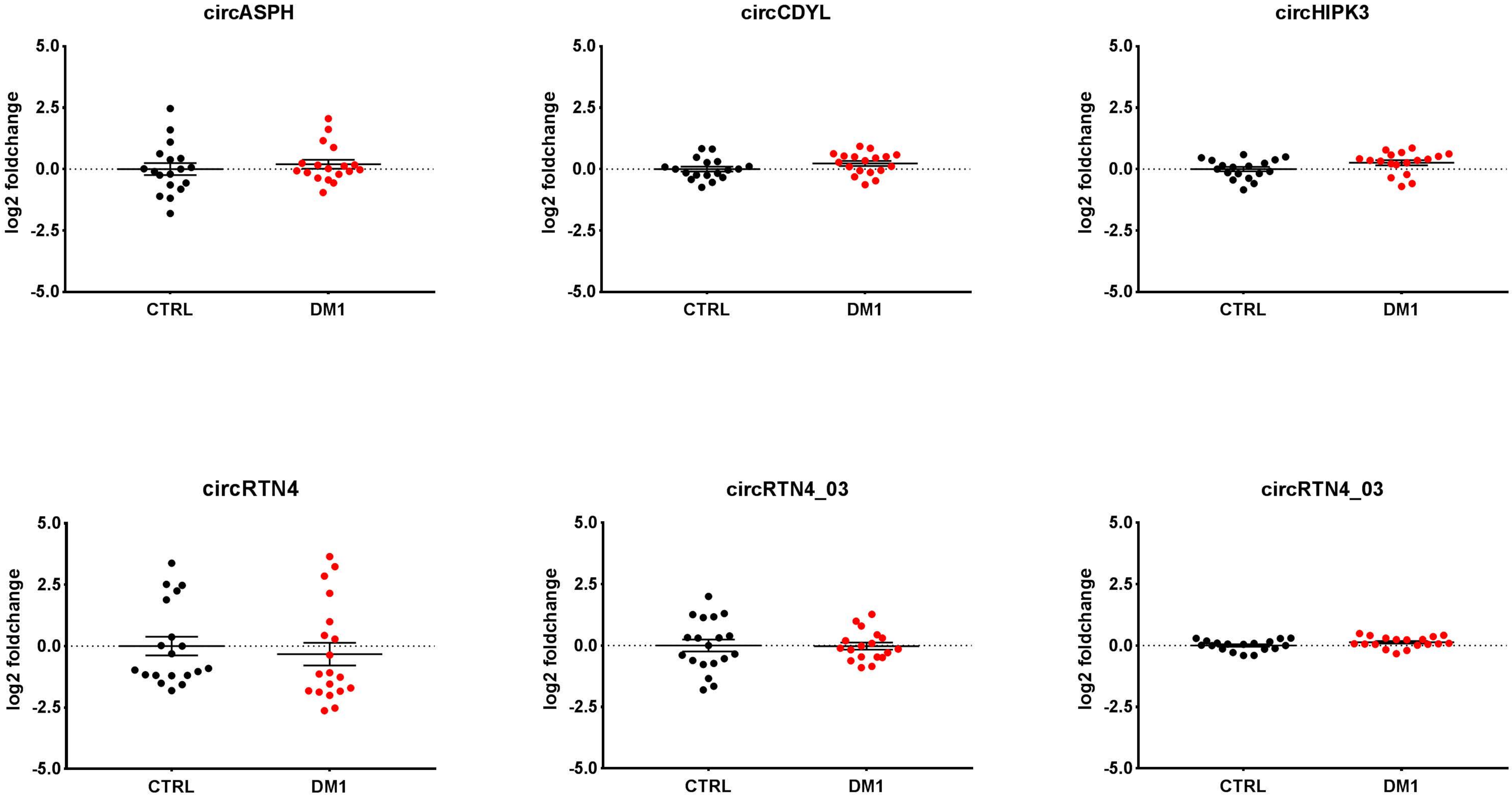

# Supplementary Figure S6

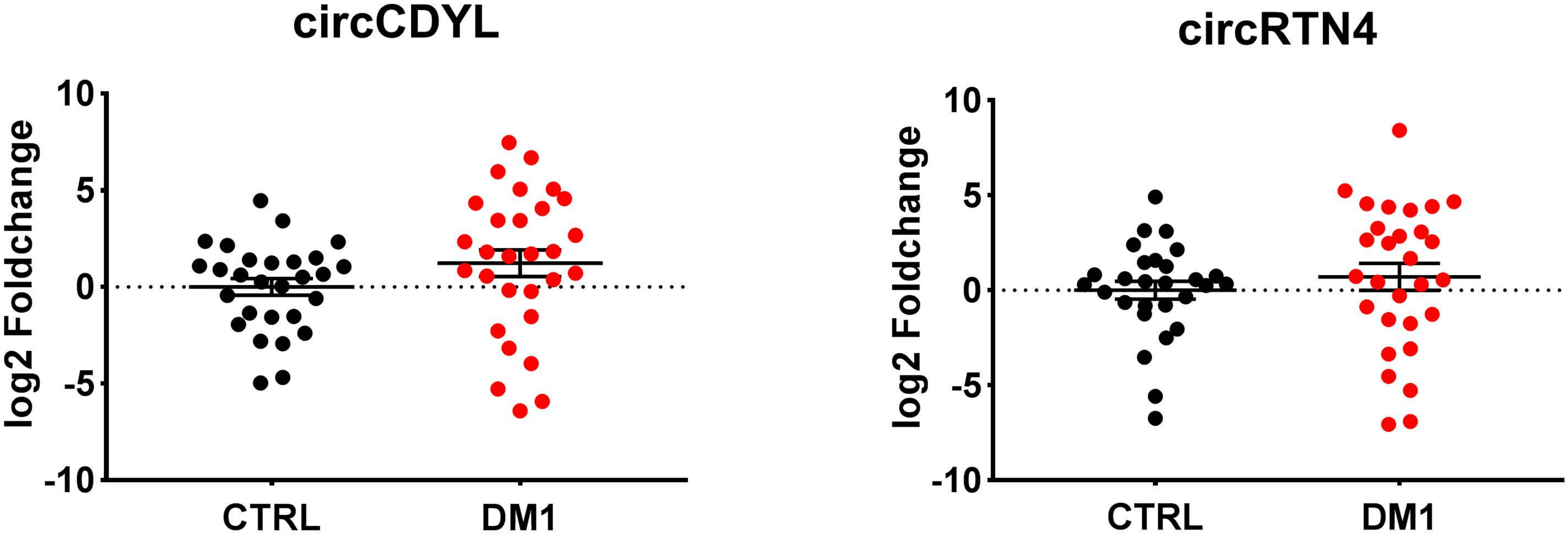

# Supplementary Figure S7

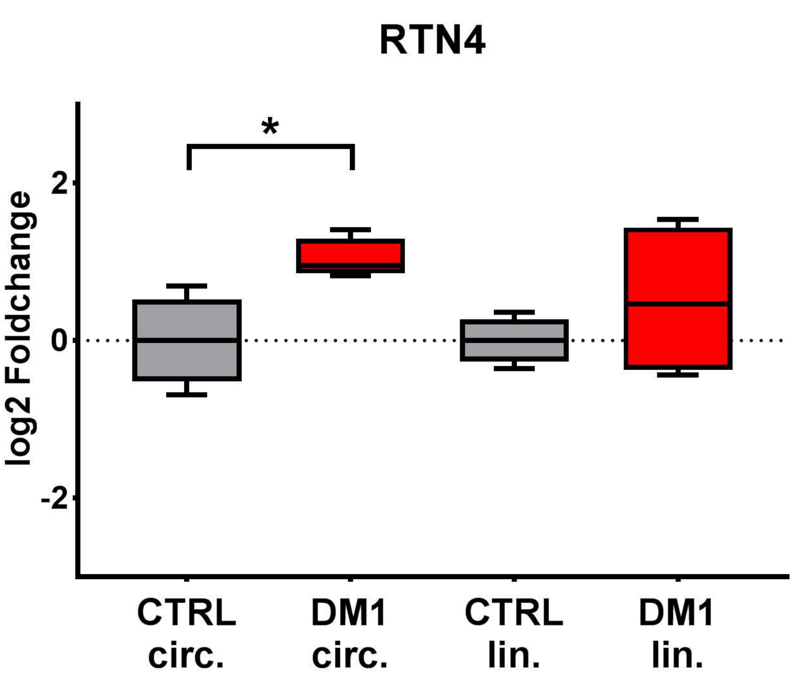


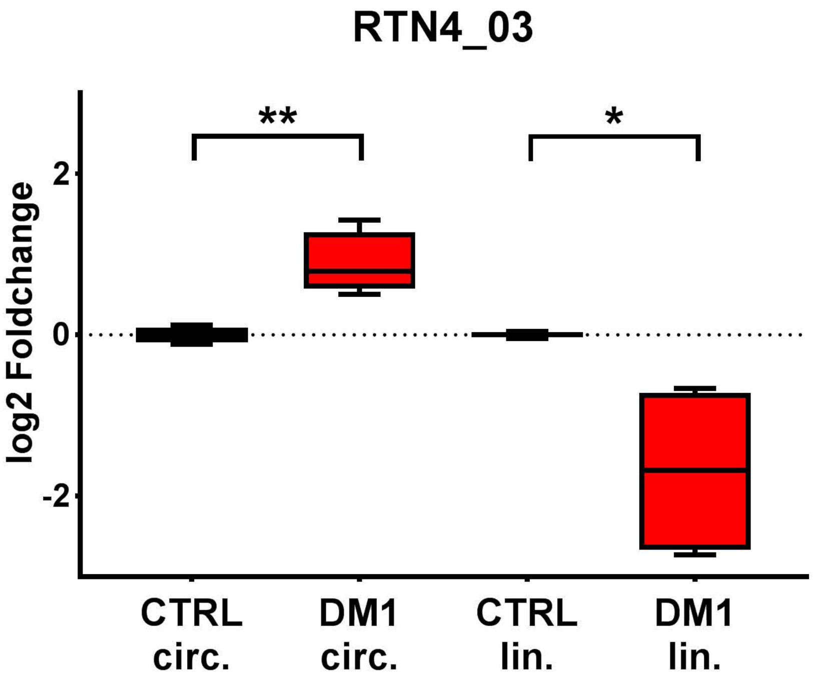

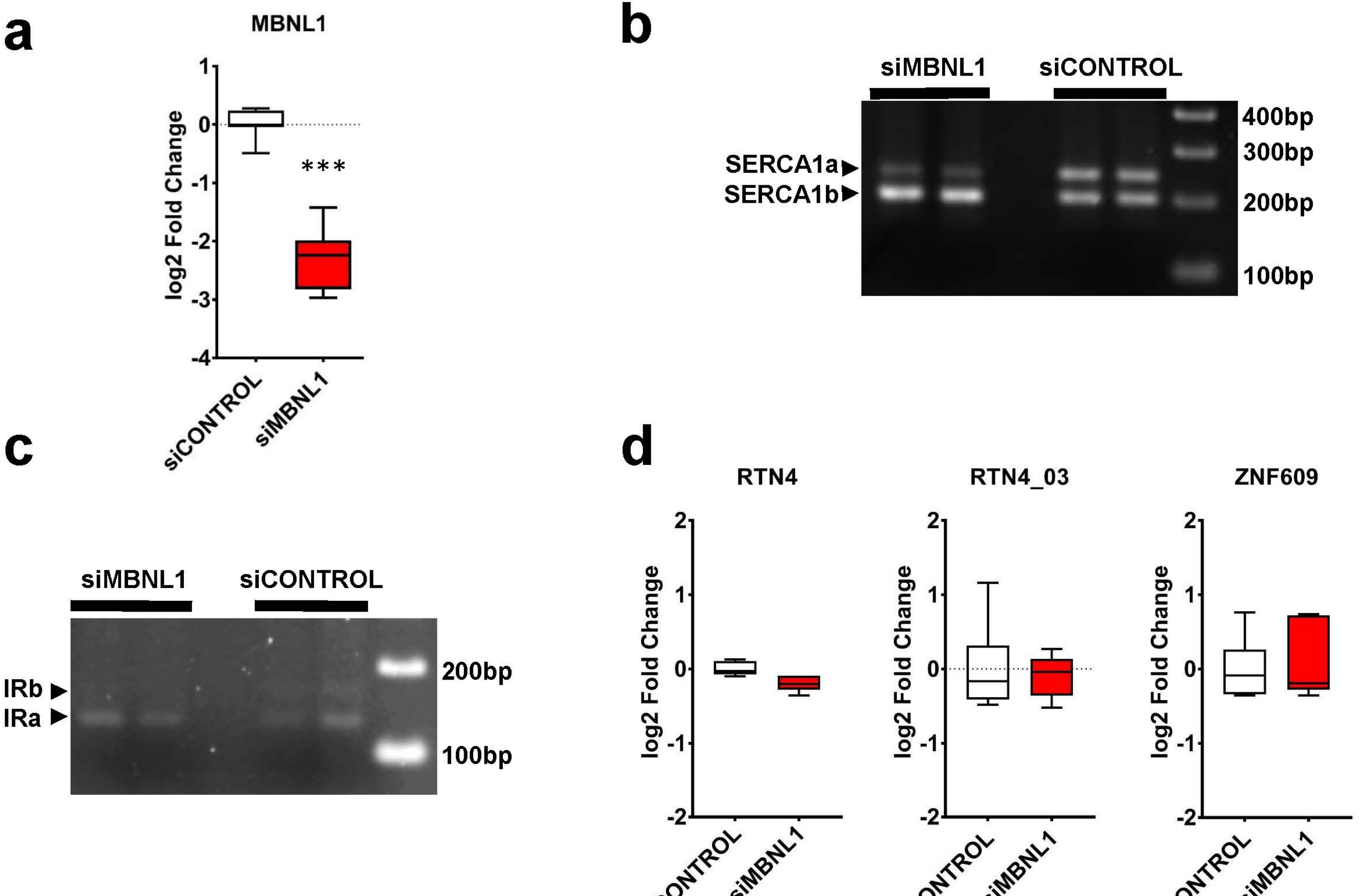


Supplementary Figure S8

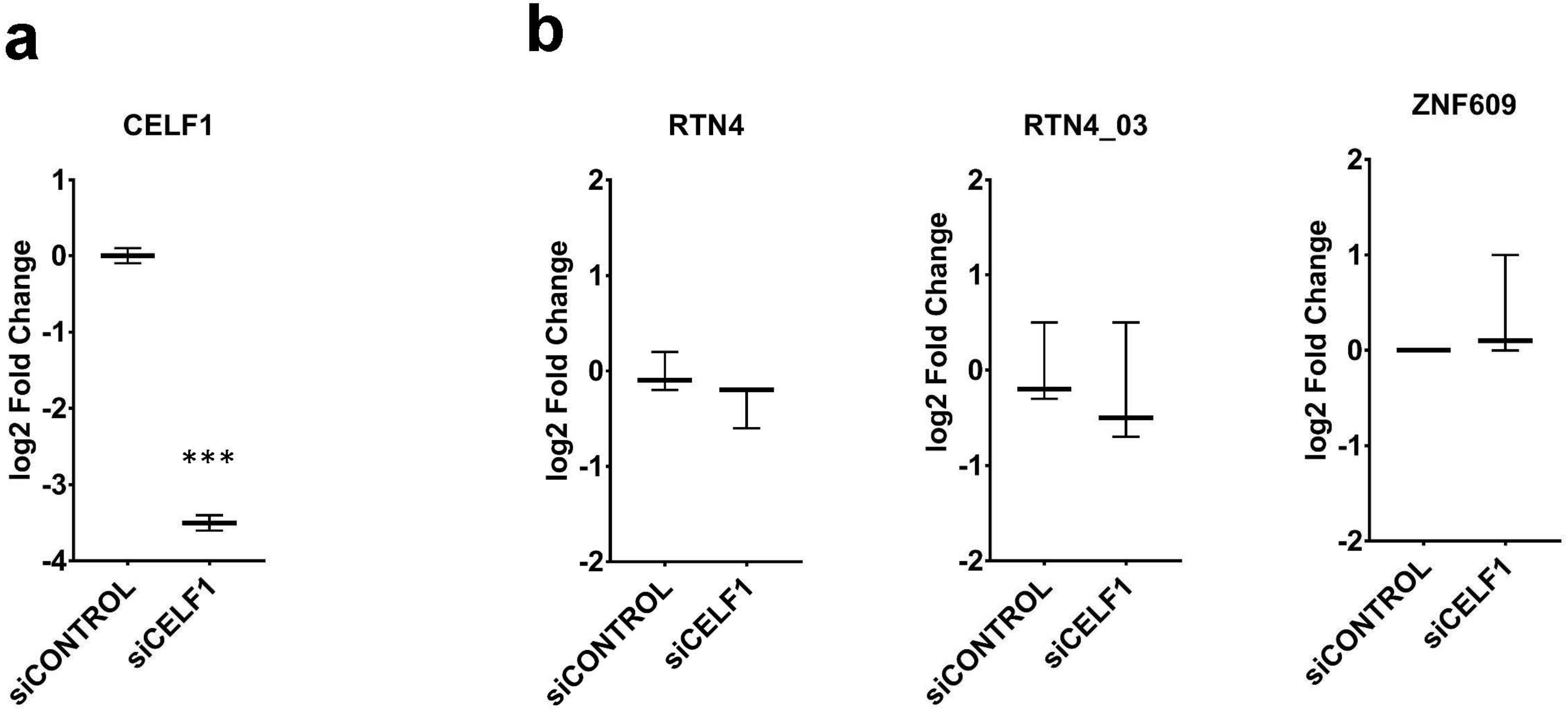


Supplementary Figure S9
