# Supplementary material for: Dysregulation of Circular RNAs in Myotonic Dystrophy Type 1
Source: Int J Mol Sci. 2019 Apr 19;20(8):1938. doi: 10.3390/ijms20081938 (PMC6515344; doi:10.3390/ijms20081938)
Supplement: Supplementary file 1 [file ijms-20-01938-s001.zip › Supplementary/Supplementary.Legends_Fig.S1-S9_TableS1-10.docx]

# Dysregulation of circular RNAs in myotonic dystrophy type 1

Voellenkle et al.

# Supporting information

## Supplementary figure legends

**Figure S1.** Histopathological analysis of biceps brachii biopsies obtained from a representative DM1 patient (a, b) and from a representative control (c). In DM1 patient, Hematoxylin & Eosin (a) and ATPase pH 10.4 (b) stainings displayed the characteristic histopathological features of DM1, such as central nuclei (asterisks), atrophic fibers (arrow) and an evident fiber size variability both of type 1 (negative fibers) and type 2 (brown fibers) fibers.

**Figure S2. Significantly modulated circRNAs in DM1 skeletal muscles.** Scatterplots of circRNA (circ.) transcripts identified by qPCR as differentially expressed in DM1 biceps brachii biopsies are shown together with their linear counterparts (lin.). Lines indicate mean and standard error values for each group. After normality test, statistical significance was calculated either by t-test or Mann-Whitney test (threshold p<0.05), followed by correction multiple comparison, with significance threshold set at q<0.01 (*q<0.01; **q<0.001). DM1= 20 (red dots), Controls= 19 (CTRL, black dots).

**Figure S3. Verification of primer specificity of DM1-circRNAs. Melting curve analysis** of primers designed for **(a)** DM1-circRNAs and **(b)** their linear counterparts. Each of the used primers displayed a melting curve with a single peak, indicating the production of a single, specific amplicon. **(c) Agarose gel electrophoresis** (2.5%) using PCR products of DM1-circRNAs. The expected size of the amplicon is shown underneath each lane. For each of the circularRNA fragments a single band of expected length and could be confirmed.

**Figure S4. Discrimination of DM1 from healthy patients by circRNA modulation.** ROC curves show the sensitivity and specificity of each DM1-circRNA to distinguish DM1 from healthy muscle tissue. DM1= 30, CTRL= 29.

**Figure S5. DM1-circRNA levels in PBMCs derived from DM1 patients and controls.** RNA was extracted from PBMCs of DM1 and control individuals. Scatterplots show the levels of DM1-circRNAs detected by qPCR. DM1= 19 (red dots), Controls= 18 (CTRL, black dots). Lines indicate mean and standard error values for each group.

**Figure S6. DM1-circRNA levels in plasma derived from DM1 patients and controls.** RNA was extracted from platelet-free plasma of DM1 and control individuals. Scatterplots show the levels of circRNAs detectable by qPCR. DM1= 29 (red dots), Controls= 28 (CTRL, black dots). Lines indicate mean and standard error values for each group.

**Figure S7. Significantly modulated circRNAs in DM1 myogenic cell lines.** Boxplots of differentially expressed circRNAs (circ.) and their linear counterparts (lin.) identified by qPCR in differentiated DM1 myogenic cells compared to controls (*p<0.05; **p<0.01; DM1= 4; CTRL= 4).

**Figure S8. MBNL1 silencing does not affect DM1-circRNA levels.** Differentiated control myogenic cells were transfected with siRNAs targeting MBNL1 or with control siRNAs (n=5). (**a**) Efficiency of MBNL1 knock-down was assessed by qPCR (*** p<0.0001). (**b** and **c**) MBNL1 silencing induced the expected increases of the SERCA1 isoform excluding exon 22 (isoform b), and of the IR isoform excluding exon 11 (isoform a), as assessed by PCR followed by agarose gel electrophoresis. Representative gels are shown. (**d**) circZNF609, circRTN4 and circRTN4_03 levels were measured by qPCR. None of the circRNAs displayed a statistically significant increase.

**Figure S9. CELF1 silencing does not affect DM1-circRNA levels.** Differentiated DM1 myogenic cells were transfected with siRNAs targeting CELF1 or with control siRNAs (n=3). (**a**) Efficiency of CELF1 knock-down was assessed by qPCR (*** p<0.0001). (**b**) circZNF609, circRTN4 and circRTN4_03 levels were measured by qPCR. None of the circRNAs displayed a statistically significant decrease.

## Supplementary table legends

**Table S1.** **Library size of publicly available data-sets used for circRNA identification in DM1 skeletal muscle by RNAseq**. A set of 30 transcriptomes (25 DM1 and 5 healthy controls) from human tibialis biopsies (GSE86356) was investigated for back-splice events. The sequencing depth is reported as million sequenced reads after mapping to the human genome version hg19. Each data-set is identified by its SRA Run number; controls are highlighted in grey.

**Table S2. circRNA identification and expression in DM1 skeletal muscle by RNAseq.** Normalized counts of 1797 different circRNA species identified by CIRI2-algorithm, following a filtering step for abundance. Each back-splice junction is identified with the coordinates of the involved donor and acceptor sites in the format “chromosome number: donor position | acceptor position”. Each data-set is identified by its SRA Run number, controls are highlighted in grey.

**Table S3. Ratios of circular versus linear expression levels measured in DM1 skeletal muscle by RNAseq.** For estimation of circular-to-linear ratios, the linear junction with the highest coverage involved with either the donor or the acceptor site of the back-splice event was determined. The averaged, normalized counts across all libraries in each condition were calculated for linear and back-splice junction. The circular-to-linear ratios were determined for controls and DM1 and are here highlighted in grey. Additionally, the circRNAs identified in human tibialis biopsies were intersected with myogenic circRNAs identified by Legnini et al. [27] The final validation set chosen for qPCR is displayed in the column “Validation-set”.

**Table S4. Primer-sequences of validation-set and alternative splicing quantification.** List of primer-couples used for relative quantification of circRNAs and their linear counterparts by qPCR. With the exception of CDYL and HIPK3, circular transcripts and their linear counterparts shared one primer, either forward or reverse.

**Table S5. Relative expression levels of circular and linear transcripts in skeletal muscle biopsies of DM1 patients.** Raw Cts derived by SYBR green qPCR were averaged across all DM1 (n=30). Means obtained by RNAseq, averaging the normalized counts of 25 DM1 libraries.Table S6. Correlation of PSI values of known DM1 alternative splicing events with expression of DM1-circRNAs in same tibialis anterior biopsies.

**Table S6. circRNA identification and expression in DM1 quadriceps muscle by RNAseq.** Normalized counts of DM1 circRNA identified by CIRI2-algorithm. Each back-splice junction is identified with the coordinates of the involved donor and acceptor sites in the format “chromosome number: donor position | acceptor position”. Each data-set is identified by its SRA Run number, controls are highlighted in grey.

**Table S7. Ratios of circular versus linear expression levels identified by RNAseq in DM1 quadriceps.** For estimation of circular-to-linear ratios, the linear junction with the highest coverage involved with either the donor or the acceptor site of the back-splice event was determined. The averaged, normalized counts across all libraries in each condition were calculated for linear and back-splice junction. The circular-to-linear ratios were determined for controls (highlighted in grey) and DM1. CircRNAs passing the abundancy filter are highlighted in yellow.

**Table S8. Correlation of PSI values of known DM1 alternative splicing events with expression of DM1-circRNAs in same tibialis anterior biopsies.**

**Table S9. Correlation of the percentage of exon usage of three known DM1 alternative splicing events with circ/lin ratio of DM1-circRNAs in biceps brachii biopsies.**

**Table S10. Clinical data of DM1 patients and controls used for circRNA detection in plasma and PBMCs.** NR: not relevant, NA: not available.
